# Silicon Integrated Interferometric Optical Gyroscope

**DOI:** 10.1038/s41598-018-27077-x

**Published:** 2018-06-08

**Authors:** Beibei Wu, Yu Yu, Jiabi Xiong, Xinliang Zhang

**Affiliations:** Wuhan National Laboratory for Optoelectronics, Huazhong University of Science and Technology, Wuhan, 430074 China

## Abstract

Miniaturized and low-cost optical gyroscopes are urgently required for emerging applications in consumer electronics market. In this paper, we proposed a theoretical analysis and preliminary experiment results for integrated interferometric optical gyroscope based on the silicon-on-insulator (SOI) platform for the first time. The gyroscope is based on the Sagnac effect and composed of coiled multimode waveguides to reduce propagation loss and the footprint. The sensitivity of the sensing part is fully investigated in terms of waveguide loss, gyroscope footprint, crossing numbers for coiled waveguides, as well as the waveguide cross section. The experimental results show that gyroscope sensitivity is 51.3 deg/s with a footprint of 600 μm × 700 μm.

## Introduction

Gyroscope is a kind of angular velocity sensor, which is widely used on airplanes, ships, cars and even mobile phones nowadays. Majority of high-precision gyroscopes are based on the optical Sagnac effect^1^ such as ring laser gyroscopes (RLGs)^2,3^ and interferometric fiber optic gyroscopes (IFOGs)^4–6^, with sensitivities in the range of μdeg/s to thousand μdeg/s. However, RLGs and IFOGs are expensive bulk sensors, resulting in the disadvantages of weight, footprint and power consumption. Because of ever increasing demands for gyroscope in the consumer electronics market, the major concern is on miniaturization and low cost. Micro electro-mechanical system (MEMS) gyroscopes^7^ have gained a lot of attention for the small size. However, the MEMS gyroscopes suffer from the unreliability caused by the unstable mechanical structure. Since their sensing elements include moving parts, an acceleration or vibration will be mistakenly detected as the angular speed^8^. Furthermore, the MEMS gyroscopes need special packing and complex readout electronic circuit, which increase the costs. Integrated photonics is the future development trend for communication and sensing system. Compared with RLGs, IFOGs and MEMS gyroscopes, chip-scale integrated optical gyroscopes have attracted increasing research attentions and could have great applications in low-cost consumer electronics market^9^.

There are many promising platforms for integrated photonics such as Indium phosphorus (InP), silicon nitride, silica, silicon-on-insulator (SOI) and so on. The researches on single and spiral high-Q resonators for InP based ROGs proved Q factor with the magnitude of 10^5^ and potential sensitivities around several 10^−3^ deg/s^10–12^. However, the InP platforms are expensive and not compatible with complementary metal-oxide-semiconductor (CMOS) fabrication technology. The specially designed ultralow loss silicon nitride interferometric optical gyroscopes (IOGs) were proposed, and a 0.025 dB/cm propagation loss was achieved^13,14^. Moreover, the silica resonator with a spiral configuration for gyroscope showed a Q factor of 1.5 × 10^6^ and a minimum sensitivity of about 2 × 10^−3^ deg/s could be expected^15^. Besides, an analysis of passive miniature resonant optical gyroscopes^16^, in terms of the propagation loss, size and power, was discussed. Nevertheless, the gyroscopes based on silicon nitride and silica planar waveguide platform are less compact than silicon for large-scale integration. Compared with silicon nitride and silica waveguides, the conventional SOI single mode waveguides have larger losses, which are bad for the performance of the gyroscopes. However, the low loss waveguide ring resonant optical gyroscopes (ROGs), with a ridged multimode structure, based on SOI were proposed with an experimental sensitivity of 27 deg/s^17^. Moreover, the dynamic optimization of a SOI-based ROG, which was coupled with Mach-Zehnder interferometer and utilized the thermos-optic effect to ensure the best sensitivity, was first reported^18^. In addition, some other schemes for better performance were investigated. For instance, an ultra-high Q CaF_2_ whispering gallery mode resonator (WGMR) based high-performance gyroscope^19^ was demonstrated, and the angle random walk about 1.7 × 10^−4^ deg/s^1/2^ was achieved. It is known that both the scattering and polarization noises impact significantly on the sensitivity degradation. A microsphere gyroscope system was established using double Faraday rotator induced orthogonal polarization to suppress the scattering and polarization noise^20^. For ring laser gyroscopes, which are expected to have better sensitivities than passive resonator gyroscopes, the lock-in effect commonly present will degrade the sensitivities. To avoid the lock-in effect in laser gyroscopes, an on-chip microresonator Brillouin gyroscope^21^ with cascaded Brillouin lasers was investigated, and a sensitivity of 6.3 × 10^−3^ deg/s is detected. To further enhance the sensitivity, Kerr nonlinearity solutions were introduced^22,23^, and a preliminary experiment results show a sensitivity of a few deg/s. Up to now, no thorough study on SOI-based IOG schemes has been reported unfortunately. Attributed to the distinct properties of SOI such as high refractive index contrast, optical transparency in C-band and the compatibility with CMOS fabrication technology, the low-cost and large-scale high density integration can be expected. However, due to the stronger light confinement in SOI platform, the characteristics such as polarization dependence, mode distribution and propagation loss are quite different from ones in other material platforms. As a result, the design and optimization of SOI-based IOGs are deserved to be comprehensively investigated.

In this paper, the destructive sensing part is selected and recorded as the representative. The coiled multimode waveguides are utilized to reduce the propagation loss and footprint. The waveguide cross section and the structure geometry are optimized. Both theoretical analysis and experimental results for optical sensing part of an integrated IOG based on SOI platform are demonstrated. A theoretical shot noise limited sensitivity of 51.3 deg/s is achieved with the sensing part in an area of 600 μm × 700 μm, and the sensitivity can be further optimized to 8.3 deg/s with an area of 2300 μm × 2200 μm, considering the given loss level.

## Results and Discussion

**Figure 1:**
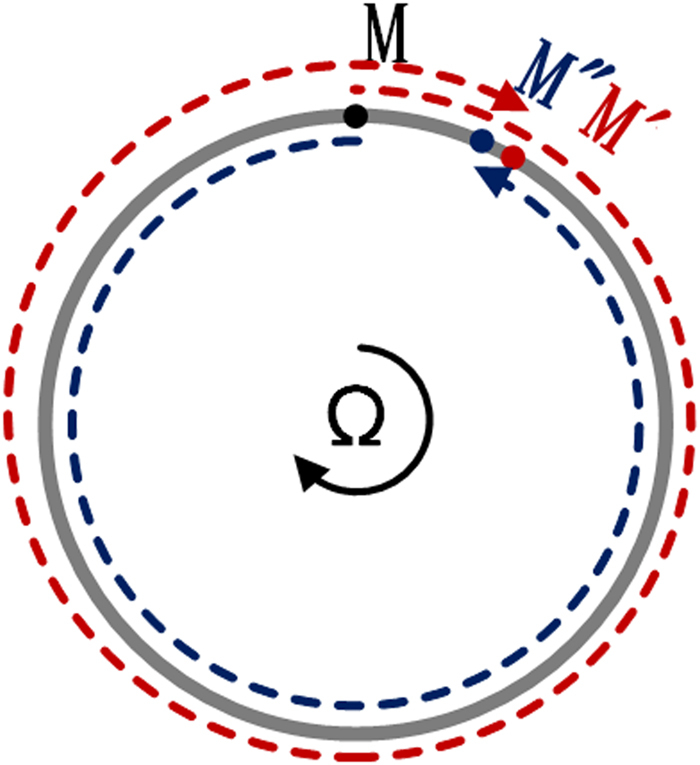
Sagnac effect in a CW rotating closed ring waveguide. Two equal beams injected from point M propagate oppositely in ring waveguide (gray solid line). After a time interval, CW beam (wine dash line) reaches displaced point M (expressed by M’). After a time interval, CCW beam (navy dash line) reaches displaced point M (expressed by M”). Under first-order approximation, point M’ and M” can regard as the same position.

Optical gyroscopes are based on the principle of Sagnac effect. For simplicity, the sensing waveguides are assumed to be circular in the following modeling. As shown in Fig. 1, light is injected into the ring waveguide via a beam splitter (at point M), and it is divided into two equal beams propagating oppositely. When the waveguide is stationary, the optical paths for the two beams are identical and they arrive at point M at the same time. If the waveguide rotates clockwise (CW) perpendicular to the plane, the CW beam experiences a relatively longer time compared with the counter-clockwise (CCW) one. The phase difference between the two beams induced by the rotation is given by

1 $${\rm{\Delta }}\varphi =\frac{8{\pi }^{2}{R}^{2}}{c{\lambda }_{0}}\Omega $$

where *R* is ring radius, *Ω* is angular velocity, *c* is the speed of light in vacuum, and *λ*_0_ is the operating wavelength. Obviously, the phase difference is proportional to the dot product of angular velocity and circular area^1^.

**Figure 2:**
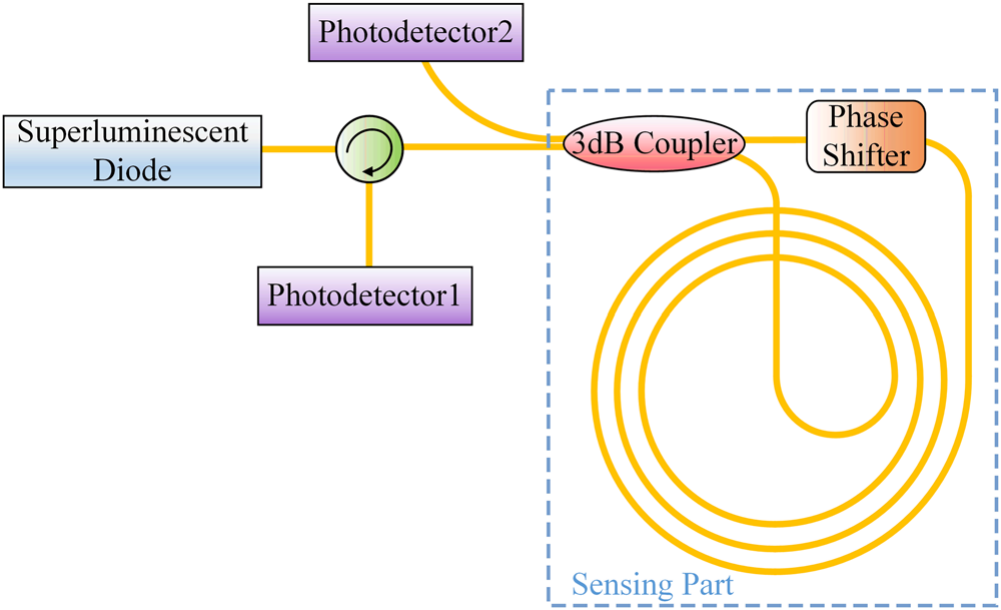
Schematic diagram of basic system of an integrated IOG.

Figure 2 shows the basic schematic of an integrated IOG system. The orange lines present optical paths. The use of broadband source such as superluminescent diodes (SLD) and superluminescent light emitting diodes (SLED) is beneficial to suppress the Kerr nonlinear effect^24^. The SLED integrated on the silicon platform using multiple die bonding and quantum well intermixing had been demonstrated^25^. The intensity-dependent Kerr effect caused nonreciprocal phase shift will be vastly suppressed with this light source. A carrier depletion phase modulator on silicon platform was demonstrated, and it can be utilized as the phase shifter in the scheme^26^. The light goes through a circulator and it is injected to a 3 dB coupler to form the CW and CCW beams in the coiled waveguides. The bi-directional beams experience different phase shift at the phase shifter. Subsequently, they interfere at the 3 dB coupler and the outputs are detected by two photodetectors, assisting by a circulator. The phase shifter is utilized to introduce a fixed *π*/2 phase difference between CW and CCW beams to reach the sensitive bias operation of the gyroscope^27^. The output powers, before detected, can be presented as,

2 $${P}_{out1}=\frac{{1}}{{2}}{a}^{2}{P}_{{in}}({1}+\,{\cos }({\Delta }\varphi +{\varphi }_{{0}}))$$

3 $${P}_{out2}=\frac{{1}}{{2}}{a}^{2}{P}_{{in}}({1}-\,{\cos }({\Delta }\varphi +{\varphi }_{{0}}))$$

where *P*_*in*_ is the incident power on the chip, *ϕ*_0_ is the fixed phase difference between CW and CCW beams, ${a}=\sqrt{{1}{{0}}^{{\rm{\alpha }}L/{10}+\gamma \cdot {2N}/{10}}}$, *α* represents the power attenuation coefficient with the unit of dB/cm, *L* is the waveguide length with the unit of cm, *γ* is the waveguide crossing loss with the unit of dB/crossing, and *N* presents the crossing number. The multiplier in front of *N* denotes that the light would go through every crossing twice. When the gyroscope is stationary, *Δϕ* = *0*. When the phase shifter works, *ϕ*_*0*_ = *π/*2, and *P*_*out1*_ = *P*_*out2*_ = 0.5·*a*^2^*P*_*in*_. Once the gyroscope rotates, the output powers will change with *Ω*.

The shot noise limited sensitivity of the integrated IOG system, represented by minimum detectable angular rate, is expressed in ref.^27^. Obviously, as mentioned in ref.^1^, the radius and length in the formula can be modified to area. The powers detected by photodetectors can be described by the form of the *P*_*in*_ attenuated in the coiled waveguides. Then, the formula can be modified as below,

4 $$\delta \Omega =\frac{c}{8AN}\sqrt{\frac{Bhc{\lambda }_{0}}{\eta \frac{1}{2}{P}_{in}\cdot {a}^{2}}}\times \frac{180}{\pi }\,(deg/s)$$

where *A* and is area, *B* is the bandwidth of the sensor, *h* is Planck constant, and *η* is the quantum efficiency. From equation (4), it can be concluded that the gyroscope sensitivity is a compromise of area and loss. Each given *α* corresponds an optimum *R* for best sensitivity. Introducing coiled waveguides to reduce area is helpful for system miniaturization but the waveguide crossings, which produce extra loss and are harmful to the sensitivity, are unavoidable. In the following, we will discuss the sensitivity and occupied area in terms of waveguide propagation loss *α*, waveguide crossing loss *γ*, the radius *R* and the crossing number *N*. The sensitivity also depends on *P*_*in*_, and a high incident power is preferred. The incident power of 10 mW, which the waveguide can withstand, is selected.

**Figure 3:**
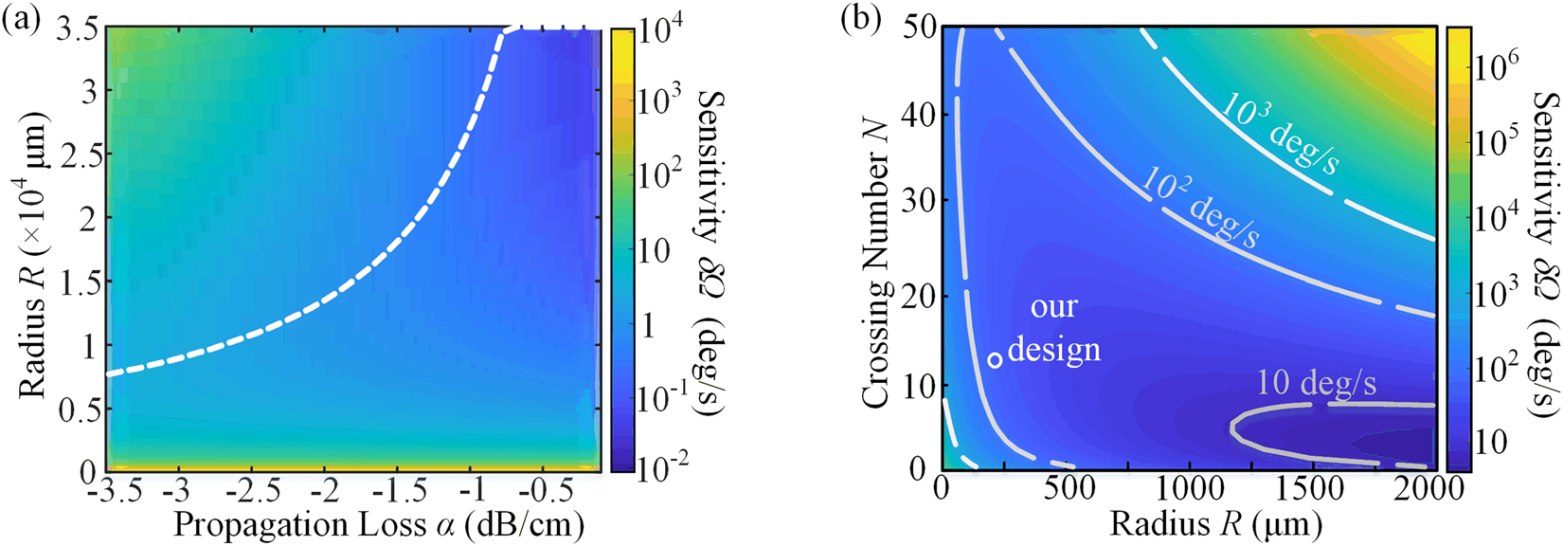
Simulation sensitivity *δΩ* of SOI based integrated IOG. (**a**) Without waveguide crossings, the calculated sensitivity *δΩ* when the values of *α* and *R* change from −3.5 to −0.5 dB/cm and from 50 to 3.5 × 10^4 ^μm. (**b**) With waveguide crossings, the calculated sensitivity *δΩ* when the values of *N* and *R* changing from 0 to 50 and 50 to 2 × 10^3 ^μm. Four graded gray lines present the contour lines.

In the following optimization simulation, the waveguide is assumed to be circular. Firstly, no waveguide crossing is involved, i.e. *N* = 1. The parameters used for simulation are listed in Table 1. Figure 3(a) shows calculated sensitivity *δΩ* with the unit of deg/s according to equation (4), when the values of *α* and *R* change from −3.5 to −0.5 dB/cm and from 50 to 3.5 × 10^4^ μm, respectively. Dashed line represents every minimum *δΩ* with different combination of *α* and corresponding optimized *R*. It indicates that the optimized *R* increases with the decrease of propagation loss. On the other hand, the smaller propagation loss gives a better sensitivity.

**Table 1:** List of simulation parameters

Symbol	Parameter	Value and Units
*c*	Light speed in vacuum	3 × 10^8^ m/s
*B*	Bandwidth	20 Hz
*h*	Planck constant	6.626 × 10^−34^ J·s
*λ*_0_	Free space wavelength	1.55 μm
*η*	Quantum efficiency	0.68
*P*_*in*_	Power incident on chip	10 mW

For high density SOI photonics, enlarging *R*, i.e. the area of the device, to achieve better sensitivity is uneconomical. The coiled waveguides are then introduced. Figure 3(b) shows *δΩ* with different *N* and *R* changing from 0 to 50 and 50 to 2 × 10^3 ^μm. A typical propagation loss of *α* = −2 dB/cm and waveguide crossing loss of *γ* = −0.045 dB per crossing are utilized in this simulation^28,29^. Three graded gray lines present the contour lines, which are marked with 10^3^, 10^2^, and 10 deg/s, in the sensitivity map. It can be noticed that in small radius region the coiled waveguides with more turn dominates, while the situation is opposite for the larger radius region where the waveguide crossing loss deteriorates the sensitivity. It is worth noting that smaller crossing loss is necessary for more crossing numbers to realize further miniaturization. Once *α* and *γ* are confirmed, the *N* corresponding to each *R* for best sensitivity can be obtained.

**Figure 4:**
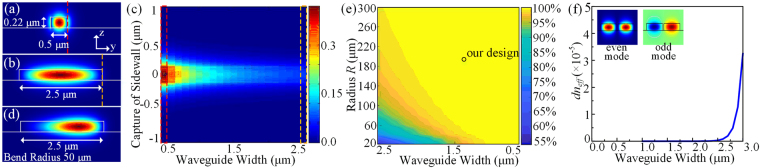
Simulation results of designed waveguide structures. Electric field component Ey of SOI waveguide (**a**) with a 0.5 μm × 0.22 μm cross section, (**b**) with a 2.5 μm × 0.22 μm cross section, (**c**) at sidewall with scanning waveguide widths from 0.5 to 2.5μm, and (**d**) with a 2.5 μm × 0.22 μm cross section of bent waveguide with radius of 50 μm. (**e**) The overlap between straight and bent waveguides with width ranging from 0.5 to 2.5 μm and radius ranging from 20 to 300 μm. (**d**) The relationship between waveguide width and d_neff_.

From the analysis above, it can be concluded that propagation loss is a conclusive factor for integrated IOGs. While the standard single mode waveguides, which are fundamental components for on-chip device, have a typical loss about −2 dB/cm^28^. The top and bottom interfaces are atomically flat in standard SOI wafers, while the etched sidewalls are not so smooth. Especially for transverse electric (TE) polarized mode, electric field intensity is much higher at the sidewall boundaries. The surface roughness of sidewall is the main source of propagation loss. Increasing waveguide width is an effective way to reduce the scattering loss. Figure 4(a) shows the simulated *E*_*y*_-field distribution of TE polarized fundamental mode in 0.5 μm by 0.22 μm cross-section SOI waveguide, which is surrounded by silica. The relatively large electric field component at the sidewall is obvious, while it can be ignored in a 2.5 μm width waveguide in Fig. 4(b). The electric field components at the sidewalls, illustrated by red and orange dashed lines in Fig. 4(a,b), are extracted and draw in Fig. 4(c) with waveguide widths scanning from 0.5 to 2.5 μm (0.1 μm step). It indicates that as the width increases, the *E*_*y*_ decreases to a small value at the sidewall, and the roughness caused scattering loss can be reduced.

Another issue needs to be taken into account is the mode mismatch between straight and bent multimode waveguides. As shown in Fig. 4(b,d), the TE fundamental modes in 2.5 μm width straight and bent (50 μm bend radius) waveguides have very different distributions. The mode field is squeezed to the outer radius in bent waveguide. In multimode waveguide, the mismatch between straight and bent waveguides excites high order modes, which are undesirable. A simulation of overlap between straight and bent waveguides with different widths ranging from 0.5 to 2.5 μm and radii ranging from 20 to 300 μm is present in Fig. 4(e). The bend radius and waveguide width both need to satisfy the relationship indicated by the brightest yellow region, where mode overlap is approaching to 100%, and mode mismatch can be ignored.

Moreover, the spacing and cross talk of long coiled waveguides need to be discussed. Ultra-low-loss waveguide arrays with spacing of 3.25 μm^29,30^ are utilized in our design. The larger waveguide width is, the smaller gap between two neighboring multimode waveguides should be with a given spacing. With small gap, cross talk must be considered. The real parts of *E*_*y*_-field of even and odd modes for two identical waveguides are shown in the insets of Fig. 4(f). The crossover length (CL) of two coupled waveguide inversely depends on the difference *dn*_*eff*_ between *n*_*eff,even*_ and *n*_*eff,odd*_ Small *d*_*neff*_ = *n*_*eff,even*_ − *n*_*eff,odd*_, which relates to a long CL, is required since infinite CL corresponds no crosstalk between neighboring waveguides^17^. Figure 4(f) indicates the relationship between waveguide width and *dn*_*eff*_ for fixed waveguide spacing of 3.25 μm. Considering the *d*_*neff*_ and gap, it can be concluded that a waveguide with width less than 2 μm is preferred.

**Figure 5:**
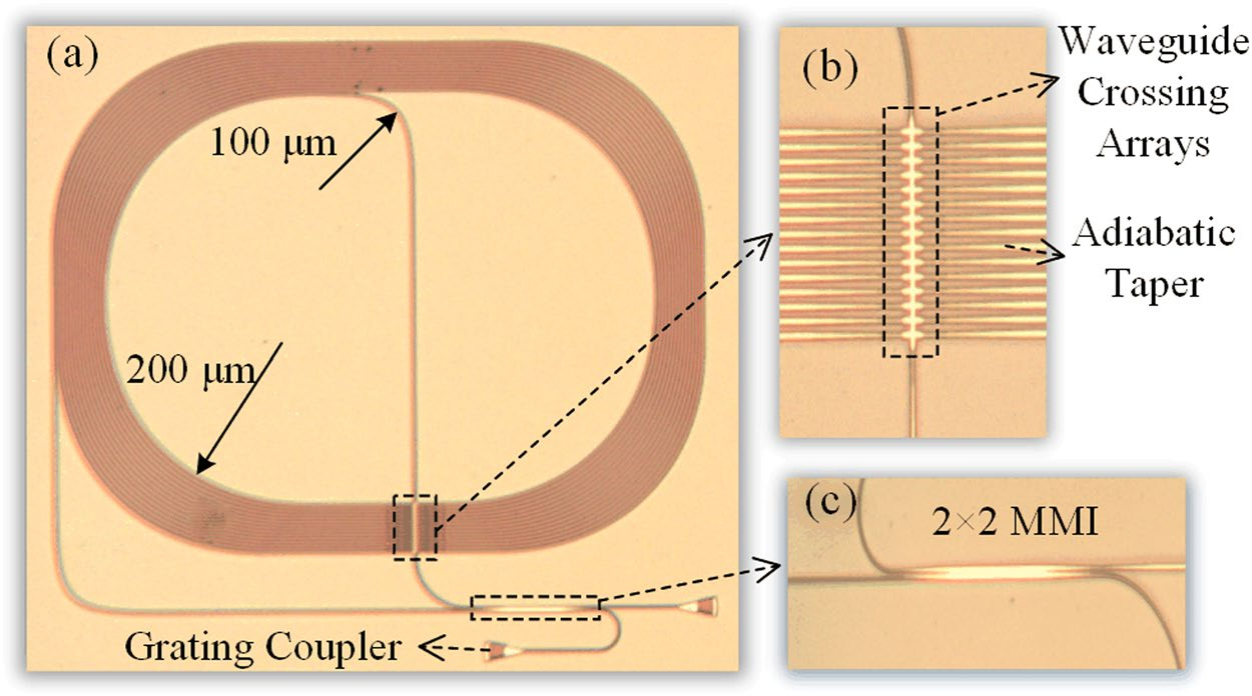
Metalloscope photos. (**a**) Full view of the device. (**b**) Detail view of MMI. (**c**) Detail view of 15 crossings.

According to the simulation discussed above, parameters for the IOG waveguide geometry are selected. The bend radius of 200 μm, crossing numbers of 15, indicated in Fig. 3(b), and waveguide width of 1.3 μm, indicated in Fig. 4(e), are utilized. The proposed passive structure is fabricated, and the metalloscope image is shown in Fig. 5(a–c) show the detailed views of 15 waveguide crossings and multimode interferometer (MMI). The grating couplers are utilized to couple light from/to light source/optical spectrum analyzer. The MMI acts as the 3 dB beam splitter. The bent and straight waveguides in the coiled waveguides are all multimode waveguides. However, waveguide crossing arrays and MMI are designed for single mode waveguides. Few 30 μm long adiabatic tapers are introduced to connect multimode waveguides with standard single mode waveguides. Therefore, the actual fabricated coiled waveguides are race track geometry. According to equation (4), further increasing ratio of radius over perimeter benefits sensitivity, i.e. the coiled waveguides approaching to circle is preferred. To form the coiled structure, the smallest radius of bent multimode waveguides is 100 μm, which has a mode overlap of 99.87% between straight and bent waveguides.

**Figure 6:**
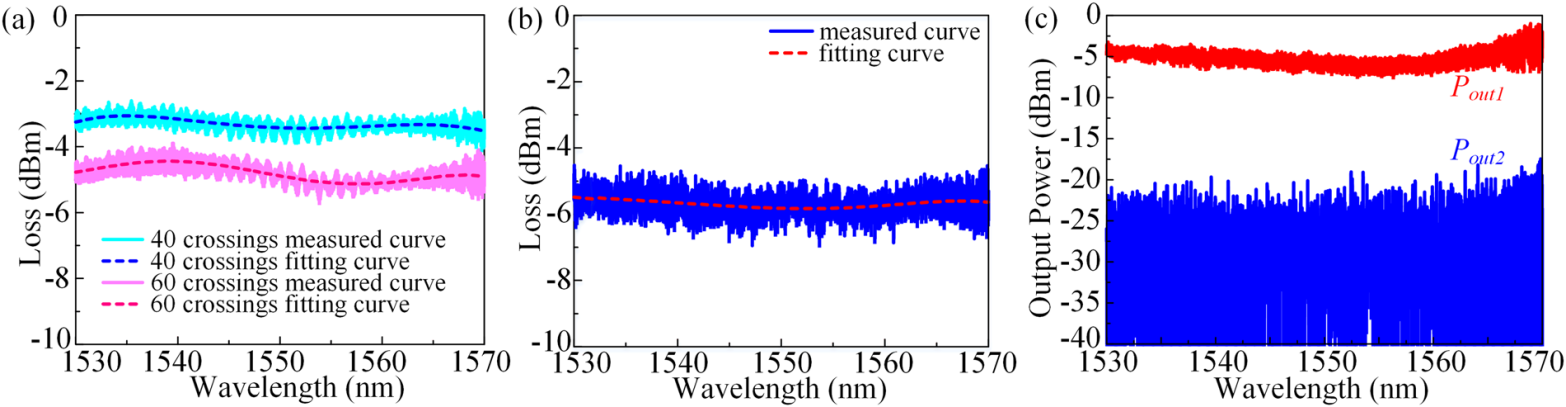
Mesurement results. (**a**) The loss spectrum of 40 crossings (cyan line), fitting curve (blue dash line), the loss spectrum of 60 crossings (magenta line), and fitting curve (pink dash line). (**b**) The loss spectrum of coiled waveguides with 15 crossings, and the fitting curve (red dash line). (**c**) Output power spectra of the sensing part (shown as *P*_*out1*_ in red and *P*_*out2*_ in blue).

For an accurate estimation, the 40 and 60 crossing arrays are fabricated on the same chip specifically for the reference measurement. The loss spectra of 40 and 60 crossing arrays are plotted in Fig. 6(a). The slope of the net loss versus crossing numbers gives the crossing loss at each wavelength. The calculated average crossing loss is −0.075 dB/crossing at 1550 nm. Then, we also fabricated the same coiled waveguides on the same chip to measure the waveguide propagation loss, without the 2 × 2 MMI. The total waveguide length is 2.76 cm and 15 crossings are included. The loss spectra of the reference device are plotted in Fig. 6(b). Subtracting the loss of 15 crossings, the average propagation loss is −1.328 dB/cm, which is much lower than ordinary fundamental mode SOI waveguide utilizing 248 nm deep ultraviolet (UV) lithography technology^31^. We repeated the measurements of different reference devices and the calculated average losses are almost the same. Figure 6(c) shows the output power spectra. It satisfies the equation (2 and 3), when the gyroscope is stationary without a phase shifter, i.e. *ϕ*_*0*_ = 0.

**Figure 7:**
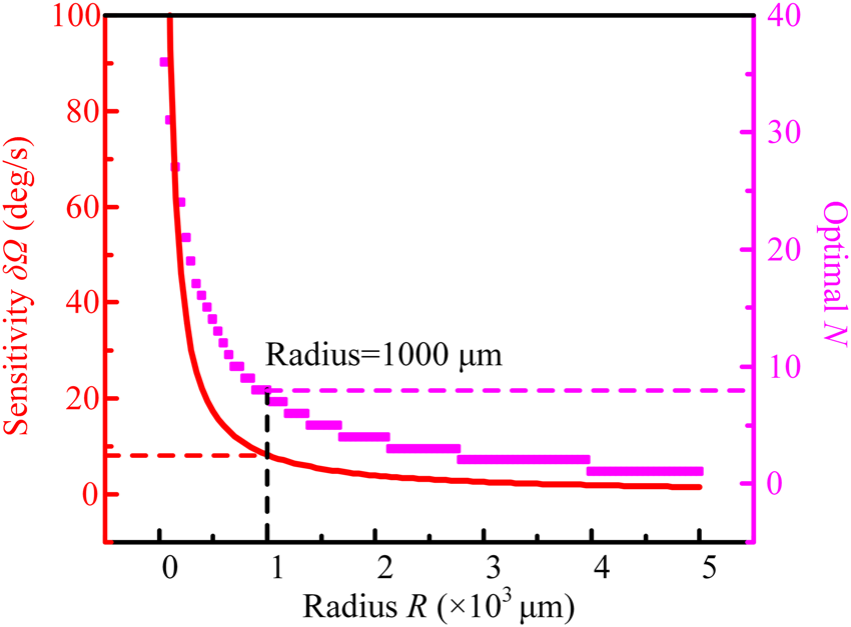
Simulation sensitivity *δΩ* and corresponding optimized crossing number *N* of SOI based integrated IOG. Propagation loss *α* = −1.328 dB/cm and waveguide crossing loss *γ* *=* −0.075 dB/crossing.

Based on the theoretical analysis and measured results, it can be calculated that the shot noise limited sensitivity of the actual fabricated sensing part is around 51.3 deg/s with a small footprint of 600 μm × 700 μm. Furthermore, with the measured propagation and crossing losses (*α* = −1.328 dB/cm, *γ* = −0.075 dB/crossing), a further optimization can be expected by increasing the radius. Considering actual waveguide structure, Fig. 7 shows the minimum *δΩ* under each radius ranging from 50 to 5 × 10^3^ μm and corresponding optimized *N* values. The area increases with square of radius, when the radius is larger than 1000 μm, the decreasing rate of *δΩ* is getting slow, and further enlarging radius is not worthwhile for miniaturized applications. A sensitivity of 8.3 deg/s can be obtained with a radius of 1000 μm, optimal *N* = 8, and a footprint of 2300 μm × 2200 μm. Further reducing the propagation loss to −0.4 dB/cm^31^, with a special ArF immersion lithography technology, and enlarging the area, the minimum detectable rotation rate of 0.045 deg/s can be expected eventually.

In conclusion, a mathematical analysis of silicon-on-insulator based integrated interferometric optical gyroscope is presented. To minimize the footprint of the device, the coiled waveguides are introduced. The sensitivity of the sensing part is investigated in terms of loss, radius, crossing number, as well as the waveguide cross section. For demonstration, the sensing part is fabricated and characterized. A shot noise limited sensitivity of 51.3 deg/s with a small footprint of 600 μm × 700 μm is estimated. A better sensitivity of 8.3 deg/s can be obtained by increasing the radius and sacrificing the footprint to 2300 μm × 2200 μm.

## Methods

### Simulation method

We utilize frequency dependence of modes solving Maxwell’s equations to calculate mode field profiles and effective index of the waveguide.

### Device fabrication

The proposed structure is fabricated on SOI platform with a 0.22 μm top silicon layer and 2 μm silica substrate. The 248 nm deep ultraviolet (UV) lithography and inductively coupled plasma (ICP) etching processes are utilized. The etched waveguides are covered by silica cladding layer, which utilizes plasma-enhanced chemical vapor deposition.
